# Dr. Hennen's Principles of Military Surgery, (3d Edition) Exhibiting a View of the Syphilitic Question, and Important Documents Illustrative of the Mercurial and Non-Mercurial Modes of Treatment Comparatively

**Published:** 1820-09-01

**Authors:** 


*Principles of Military Surgery, comprising Observations on the Arrangement, Police, and Practice of Hospitals; and on the History, Treatment, and Anomalies of Variola and Syphilis.* Illustrated with Cases, Dissections, and Engravings. By JOHN HENNEN, M.D. F.R.S.E. Deputy Inspector of Military Hospitals. Second Edition, with numerous Additions. One volume Octavo, closely printed, pp. 580, with six Plates. London and Edinburgh, 1820.

IN the Monthly Series of this Journal, we gave a full account of the first edition of the valuable work now before us. In this edition, the quantum of matter is nearly doubled, by means of concentration and small type, while the price is but little increased. As it now stands, there is no other work on Military Surgery, in the English Language, at all to be compared with Dr. Hennen's volume. This distinguished medical officer has added to an extensive stock of personal experience and observation, a vast mass of important facts and observations from his military, naval, and civil brethren, combined with a selection from the best authors of our own and other times, all bearing on military surgery in particular; but, in truth, embracing surgery in general, and elucidating all its leading principles and operations. Such a work must prove an invaluable acquisition to the practitioners in the army and navy; and, indeed, to surgeons in general, to whom we can safely recommend it as a standard production.

In the present article, we shall confine ourselves to the subject of syphilis—a subject which so much divides the sentiments of professional men, and on which Dr. Hennen, from his official situation, and his access to authentic documents, is particularly well qualified to write. Our own experience in the non-mercurial treatment of syphilis having been very limited, we shall endeavour chiefly to exhibit a full analytical view of our able author's paper, leaving it to our readers to judge and act for themselves, after weighing the evidence here adduced.

Dr. Hennen enters into a pretty extensive historical sketch of the various other remedies besides mercury, which have been extolled, from time to time, as specifics. This, he thinks, can only be accounted for, by supposing that the disease was curable by the efforts of the constitution alone; otherwise, inert and trifling medicines could never have acquired the reputation of being anti-syphilitics. But there is another point of view, in which the subject may be taken, and in this we are strongly disposed to coincide with Dr. Hennen.

"It may be, that such an alteration has been produced in it either from the effects of remedies, or by natural causes, as seriously to influence the results of our treatment at the present day. The analogy of leprosy and of scurvy, which at one time raged throughout Europe, but are now almost unknown, is strongly in favour of the supposition of a change of character in syphilis; we have also direct testimony, which shows that its symptoms have become milder and more tractable." 496.

Dr. H. quotes several authorities for this supposition; among others, the learned and indefatigable Astruc himself, who, nearly a hundred years ago, says—

"I have, by careful and repeated observation, found the venereal disease daily to grow milder; it may, perhaps, be more frequently contracted than formerly, yet its rage is less violent, its symptoms are not so many, so painful, nor so difficult to be cured; it yields more readily to remedies properly applied; and, in a word, seems by little and little to approach towards its close."*

"The following note, containing very curious matter, we shall extract from Dr. Hennen's work.

"From some extracts from the rare volume of Ruy Diaz de Isla, "Contra las Bubas," Salamanca, 155—, with which Dr. Thomson, Professor of Military Surgery, has favoured me, I find that, in no town in Europe, of one hundred inhabitants, were there fewer deaths than ten on the first appearance of syphilis among them. Now, if we recollect that the numerous monks and nuns spread the disease through all classes of society, it is not an extravagant calculation, that fifty out of the hundred were poxed. This will give us one death in five: the remaining four survivors must have owed their lives to the "methodical or rational" cure. I doubt whether the mortality was greatly less than this while the gross abuses of mercury prevailed, under the form of daubings, with four lb. of quicksilver to two lb. of lard, applied over the whole body. "Cum quo (says Torella) infinitos interfecerunt, interficiuntur homines, non moriuntur." (Luisin, tom i. p. 528.) But, putting the mortality out of the question, Hutten expressly states, that hardly one in one hundred was perfectly cured, the disease returning upon them again, as it did on himself after eleven salivations. (See Ulrichus de Hutten de Morbo Gallico apud Luisinum, cap. 4. tom. i. p. 281 and 283;) but after guaiacum was introduced, only one individual died in Germany while under its use, and his death was attributed to excess in venery." 498.

If the latter part of the above passage be true, (which we

---

* Astruc, lib. 1, cap. 13. Hennen, p. 497.

much doubt) then guaiacum, as well as syphilis, has lost some of its pristine powers.

Speaking of the opinion entertained by several of the present day, and once prevalent all over Germany, that the diseased state of the bones, in protracted syphilitic cases, is the effect of mercury, and not of the venereal virus, Dr. Hennen remarks,

"It becomes a point well worthy of inquiry, whether mercury produces diseases of the bones where a predisposition to these diseases does not exist. I am well convinced that the carious affections of the bones, which are so common in persons treated by long mercurial courses, have proceeded, not from the disease, but from the remedy rapidly and irregularly thrown in while periostitis existed; as a proof of which, I have not seen a single case of carious bone in the military hospitals since the non-mercurial treatment was adopted, except where mercury had formerly been used, so that those gentlemen who so kindly and compassionately harangued on falling noses and rotten bones, have displayed their sensibility in vain." 506.

Upon this we can only remark, that as we have seen mercury used to a great extent, and among whole masses of men of various constitutions, without these diseases of the bones supervening, we cannot but suspect that the syphilitic virus is the principal cause of such formidable affections.

The observation of Leo Africanus, nearly three hundred years ago, that lues venerea undergoes a cure by change of climate in Africa, without the employment of mercury or other medicine, is confirmed, Dr. H. thinks, by the recent traveller, Horneman; but we confess, that the extract brought forward from this gentlemen, has nothing very satisfactory for our minds.

"Dr. Mitchell, of New York, in the Medical Repository for 1799, assures us, that a great number of venereal ulcers were cured in the hospitals of that city, by the local application of potash, or salt of tartar, from which fact he draws the very natural conclusion, that neither nitrous acid nor mercury produced any *specific* effect upon these ulcers." 508.

Our author, after enumerating the various morbid affections produced by mercury, when either profusely used, or in unfavourable constitutions, observes:—

"While I have thus enumerated many of the ill effects produced by mercury when it acts as a poison, I must give my strongest testimony to the admirable results which proceed from its judicious use in persons not constitutionally disposed to be injured by it, and who do not lead profligate lives, or are not exposed to the foul air of hospitals fully saturated with its fumes." 512.—"Of its unquestionable efficacy there can be no doubt; but its indiscriminate employment in every case, whether old or recent, suspicious or confirmed, and without any view to the patient's diet, or his general health, has produced the most dreadful consequences." 513.

This, indeed, we have all along considered to be the real state of the case. The *abuse* of the medicine is to be deprecated; not its rational use. Dr. H. properly observes, that the precepts of Hunter, Abernethy, Pearson, Carmichael, and several others, have reduced the quantity of mercury formerly given in syphilitic affections.

"But it remained for the inquiry which is at present prosecuting in the military hospitals, to show, that even these bounds are too extensive, and that the practitioner has, in a vast number of instances, the option whether to defer its use, to limit it, or to omit it altogether. Settled as it now is, beyond a doubt, that syphilis does not run on uninterruptedly to a fatal event, if not checked by mercury, that practitioner cannot be admitted to do full justice to his patient, who does not avail himself of the fact—to his own judgment must be left the extent to which he may be pleased to do so."

Dr. Hennen candidly confesses his inability to point out "any invariable characteristic symptom by which to discriminate the real nature of the primary sore," while "we are equally at a loss in many of the secondary symptoms."

"It would be by no means difficult to show that the high round edge, the scooped or excavated sore, the preceding pimple, the loss of substance, the hardened base and edge, whether circumscribed or diffused, and the tenaciously adhesive discharge of a very fetid odour, are all observable in certain states and varieties of sores unconnected with a venereal origin. The hardened edge and base, particularly, can be produced artificially, by the application of escharotics to the glans or penis of a sound person; and if any ulceration or warty excrescence previously exists on these parts, this effect is still more easily produced." 517.

What, then, are the practical steps to pursue in diseases produced by sexual intercourse? We shall lay the precepts of our able and experienced author before the reader.

"1st. In every primary ulcer, I would give up the idea of using mercury at first, treating it as if it were a simple ulceration, by cleanliness, rest, and abstinence, and applying to it the most simple and mildest dressings.* If the sore did not put on a healing appearance in a reasonable time, the extent of which must depend on the circumstances of the patient, I should make use of more active dressings. But if, beyond all calculation, it remained open, I should certainly not sacrifice every consideration to a dislike of mer-

* A very early application of lunar caustic will often supersede all other remedies.

cury, knowing how many persons have been seriously benefited by a judicious and mild administration of that remedy. 2d. The same principles which guide me in the primary ulcers, would have the same, if not greater force, in the case of buboes. In their irritable state, I consider mercury as altogether inadmissible. Moderate pressure, and cold applications, will often disperse them in their commencement, aided by Girtanner's plan of frictions of volatile liniment on the thigh of the affected side. If they suppurate, opening with the kali purum is by far the best plan; they then heal from the bottom. 3d. The cutaneous eruption I would treat at first on the same general principle, but I should not very long postpone the employment of the mildest mercurial alteratives, aided by warm bathing and sudorifics. 4th. In the affections of the throat I would be more guarded than in any others in the employment of mercury, until all inflammatory disposition was removed; after that, I have seen them yield, as if by magic, so soon as the local effects of the mercury on the parts within the mouth became obvious; but before the inflammatory symptoms were subdued, I have seen a vast number of instances where irremediable mischief has been done. 5th. In the bone cases, during the stage of periostitis, or any approach towards it, local bleeding, blistering, warm bathing, and abstinence, are the proper remedies, to the entire exclusion of mercury. When inflammation is subdued, that remedy may be tried in moderation; but if caries exist, I hold it to be highly improper under any form." 519.

Our author states, that so far has delay been from injurious, in the cases under his inspection, that the sanative powers of mercury were remarkably assisted by the preparatory regime of cleanliness, rest, rigid abstinence, purgatives, sudorifics, and venesection, where deemed necessary. This was particularly remarkable where the genital ulcerations were irritable and extensive, threatening the destruction of the organ; or where the throat was severely affected. The extent of the delay in administering mercury, must depend on the individual case. In delicate phthisical habits there is no necessity for haste.

"At the same time, no person in private practice, whose constitution could tolerate mercury, would willingly continue to bear about his person those suspicious-looking stains and eruptions on the skin, which a judicious employment of that remedy so often relieves." 519.

While, therefore, great pains were taken to ascertain how far mercury could be safely dispensed with in the treatment of syphilis, no patient's constitution was involved, nor practitioner's character compromised in the experiment.

"In no case, (says Dr. Hennen) I most firmly believe, has the health of an individual been wantonly trifled with, nor has the utmost professional exertion been spared to elucidate the history of this most interesting and most common of all military diseases, even in the persons of some of the professional men themselves."

This, we have the strongest reason to believe, is strictly fact. That any *external* applications of mercury could have had any constitutional effect on the disease, is quite untenable; indeed, cold water alone was used by a great many of our military surgeons. It is well known that the appearance of genital ulcerations is much modified by their locality. This should be always borne in mind. The following circumstances were generally remarked in the military hospitals.

"1. Ulcers on the external integuments have generally had round callous edges, level surfaces, but little induration of base; they were less irritable than others, became sooner clean, and healed uniformly, but slowly. 2d. Ulcers on the internal membrane of the prepuce have been generally either superficial or elevated; their surfaces covered with a light-coloured slough, or of a bright red, with a villous appearance; their edges either regularly defined, or spread out like excoriations; their bases have been, in general, but little indurated, but when the ulcers have spread out, they have sometimes acquired a cartilaginous hardness, and have been extremely difficult to heal. 3d. Ulcers immediately behind the corona glandis have been, in general, highly irritable, deep, scooped, indurated in their edges and base, foul, with membranous bridles, running across them, throwing off a perceptible slough, but, if mildly treated, soon healing after that event. 4th. Ulcers on the frœnum have generally followed lacerations of that part, have had considerable induration of base, and have been generally slow of healing. 5th. Ulcers of the glans have been generally excavated, but with little hardness of base, quickly throwing off a slough, and then healing rapidly." 524.

Dr. Hennen mentions a curious fact, which should be held in memory, especially by the military and naval surgeon. It is this—that the hardened edge and base *naturally* attending some ulcerations "may be artificially produced by the application of the kali purum to a sound man;" and this sore "is not to be distinguished from chancre by a person not acquainted with the circumstance." Even the specific distance (as it has been called) of the hardness can be increased or diminished, by proper management of the caustic.

Dr. Hennen finally comes to the conclusion, that primary sores on the genitals, of whatever description they may be, can be healed without the employment of mercury; nor has he met with any thing to make him question the propriety of making the trial. The same observation, to a certain extent, is applicable to the secondary symptoms that have succeeded the non-mercurial treatment; but here our author cannot offer such positive testimony to the expediency of abstaining from mercury altogether.

"The facts at present ascertained are these:—Secondary symptoms occur more frequently, and appear at an earlier and more determinate period than when mercury had been used; but they, in many cases, have gone off as soon, never, as has been supposed, proceeding from bad to worse, or from one succession of parts to another in unabated violence; on the contrary, they by no means exhibit the same violent and unrelenting symptoms which we have observed, in many instances, where mercury has been used; the eruptions have not run into ulceration; they have not formed into large scales or extensive blotches; nor have the bones of the nose, or of other parts, been in any instance affected with caries. I cannot take upon me to assert, that these events *will not* occasionally take place, but in the numerous cases which I have watched with the utmost anxiety, I can aver that they *have not*." 527.

Eruptions on the skin, Dr. Hennen thinks, have been more frequent since the non-mercurial treatment has been in use; but the general health did not appear to suffer more than when similar eruptions succeed the mercurial treatment, and they all gradually, though sometimes slowly, disappeared without the use of mercury. In some cases of the cutaneous eruptions, Dr. H. observed considerable benefit from the mineral acids, used both internally and externally. In no case were the bones of the nose affected, though occasional instances of periostitis, and of pains and swellings of the bones of the cranium, and of the extremities, were met with. Only two *unequivocally syphilitic* nodes were observed. One of these gave way to blisters and sarsaparilla; the other was treated with mercury.

We shall now introduce a series of analytical tables, drawn by our most intelligent and indefatigable author, from the practice of the regimental surgeons, in those corps of the army which were quartered in Scotland, during a considerable portion of the time that he held the superintendence of that part of the united kingdom.

Analytical Return of Venereal Diseases, treated without Mercury, in the Military Hospitals of Scotland, under the Superintendence of John Hennen, M. D. Deputy-Inspector, from June 20th 1817 to December 20th 1819.

### PRIMARY AFFECTIONS.

### 1. *Description of Cases that have been Treated.*

| | | | | | |
|---|---|---|---|---|---|
| A. Affections possessing the true Hunterian character. | *a.* Ulcers only | | | 109 | |
| | *b.* Buboes succeeding to ulcers. | α. Before admission into hospital | 48 | | |
| | | β. After admission into hospital | 10 | | |
| | | γ. Of which were discussed | 42 | 58 | |
| | | δ. Of which suppurated | 15 | | |
| | | ε. Under cure at the date of last return | 1 | | 167 |
| B. Affections of various kinds not possessing the true Hunterian character. | *a.* Ulcers only | | | 154 | |
| | *b.* Buboes succeeding to ulcer. | α. Before admission into hospital | 72 | | |
| | | β. After admission into hospital | 12 | 84 | |
| | | γ. Of which were discussed | 53 | | |
| | | δ. Of which suppurated | 31 | | |
| | *c.* Buboes without previous ulcer | | | 2 | 240 |
| Total number of Primary Affections treated | | | | | 407 |

### 2. *Time required for the Cure.*

#### A. of ulcers.

| Hunterian. | | | Non-Hunterian. | | |
|---|---|---|---|---|---|
| The following number of cases were cured. | In the following number of days | | The following number of cases were cured. | In the following number of days | |
| 36 | From 5 | to 10 | 63 | From 3 | to 10 |
| 19 | 11 | 20 | 40 | 11 | 20 |
| 42 | 21 | 30 | 43 | 21 | 30 |
| 13 | 31 | 40 | 10 | 31 | 40 |
| 13 | 41 | 50 | 5 | 41 | 50 |
| 5 | 51 | 60 | 1 | 51 | 60 |
| 1 | 71 | 80 | 1 | 61 | 70 |
| 2 | 91 | 98 | 2 | 71 | 80 |
| 4 under cure at the date of last return. | | | 4 | 81 | 90 |
| | | | 1 | 93 | |
| | | | 1 | 109 | |
| | | | 1 | 113 | |
| | | | 18 under cure at date of last ret. | | |

### B. OF BUBOES ENDING IN RESOLUTION.

| Buboes following Hunterian Ulcers. | | Following Non-Hunterian Ulcers. | |
|---|---|---|---|
| The following number of cases were cured. | In the following number of days. | The following number of cases were cured. | In the following number of days. |
| 5 | From 5 to 10 | 10 | From 2 to 10 |
| 8 | 11 20 | 18 | 11 20 |
| 15 | 21 30 | 13 | 21 30 |
| 4 | 31 40 | 4 | 31 40 |
| 8 | 41 50 | 4 | 41 46 |
| 2 under cure at the date of last return. | | 4 under cure at the date of last return. | |

### C. OF BUBOES ENDING IN SUPPURATION.

| Buboes following Hunterian Ulcers. | | Buboes following Non-Hunterian Ulcers. | |
|---|---|---|---|
| The following number of cases were cured. | In the following number of days. | The following number of cases were cured. | In the following number of days. |
| 2 | From 30 to 40 | 1 | From 7 to 10 |
| 3 | 41 50 | 2 | 11 20 |
| 2 | 51 60 | 5 | 21 30 |
| 2 | 61 70 | 8 | 31 40 |
| 1 | 77 | 4 | 41 50 |
| 1 | 92 | 3 | 51 60 |
| 4 under cure at the date of last return. | | 1 | 71 80 |
| | | 3 | 91 100 |
| | | 2 | 101 110 |
| | | 2 | 111 113 |
| | | 1 | 179 |
| | | 4 under cure at the date of last return. | |

*Note.* In the Table A of Ulcers, the period of cure of some of these affections is not specified, as it was not fully ascertained from some corps; but in no case did it extend beyond the average period of the cases which are specified in the Table.

### SECONDARY AFFECTIONS.

### I. *Description of Cases treated.*

| | | | | | |
|---|---|---|---|---|---|
| A. Succeeding the Hunterian ulcer. | *a.* Eruptions only | α. Tubercular | 6 | 15 | |
| | | β. Exanthematous | 5 | | |
| | | γ. Pustular | 2 | | |
| | | δ. Tubercular and Scaly | 1 | | |
| | | ε. Tubercular and Vesicular | 1 | | |
| | *b.* Eruptions combined with sore throat. | α. Tubercular | 2 | 4 | |
| | | β. Tubercular and Scaly | 1 | | |
| | | γ. Tubercular and Exanthematous. | 1 | | |
| | *c.* Tubercular Eruption combined with Iritis | | | 1 | |
| | *d.* Tubercular and Papular Eruption, combined with Iritis, Periostitis, and Sore Throat | | | 1 | |
| | *e.* Tubercular and Exanthematous Eruption, combined with Periostitis | | | 1 | |
| | *f.* Sore Throat only | | | 1 | |
| | *g.* Exostosis only | | | 1 | |
| | | | | | 24 |
| B. Succeeding ulcers not Hunterian. | *a.* Eruptions only. | α. Pustular | 6 | 12 | |
| | | β. Exanthematous | 3 | | |
| | | γ. Tubercular | 2 | | |
| | | δ. Tubercular and Scaly | 1 | | |
| | *b.* Eruptions combined with sore throat. | α. Exanthematous | 3 | 7 | |
| | | β. Tubercular | 2 | | |
| | | γ. Papular, Scaly, and Tubercular | 1 | | |
| | | δ. Pustular and Tubercular | 1 | | |
| | *c.* Sore throat only | | | 3 | |
| | | | | | 22 |
| Total number of Secondary Affections treated | | | | | 46 |

### II. *Period of Occurrence, and Time required for the Cure.*

| Description of Primary Affections to which they succeeded. | No. of Cases. | Description of Secondary Affection. | Period of occurrence after Primary Affection. | Time required for the Cure. |
|---|---|---|---|---|
| A. Succeeding the Hunterian Ulcer. | 6 | Tubercular Eruption - - - - - | From 7 ds to 6 ms | From 10 to 60 ds |
| | 5 | Exanthematous Eruption - - - | —— 28 ds to 6 ms | —— 3 to 42 ds |
| | 2 | Pustular Eruption - - - - - - | —— 21 ds to 38 ds | —— 18 to 43 ds |
| | 1 | Tubercular and Scaly Eruption | 40 ds | 25 ds |
| | 1 | Tubercular and Vesicular Eruption - - - - - - - - - - - - - | 22 weeks | 27 ds |
| | 2 | Tubercular Eruption with Sore Throat - - - - - - - - - - - | —— 46 to 75 ds | —— 31 to 240 ds |
| | 1 | Tubercular and Scaly Eruption with Sore throat - - - - - - | 5 weeks | 66 ds |
| | 1 | Tubercular and Exanthematous Eruption with Sore Throat - | 2 ms | 60 ds |
| | 1 | Tubercular Eruption combined with Iritis - - - - - - - - - | 14 weeks | 45 ds |
| | 1 | Tubercular and Papular Eruption, combined with Iritis, Periostitis, and Sore Throat | 45 ds | 84 ds |
| | 1 | Tubercular and Exanthematous Eruption, combined with Periostitis - - - - - - - - - - - | 5 ms | 28 ds |
| | 1 | Sore Throat only - - - - - - - | 5 weeks | 33 ds |
| | 1 | Exostosis only - - - - - - - - | 74 ds | 14 ms |
| B. Succeeding Ulcers not Hunterian. | 6 | 5 Pustular Eruption* - - - - | From 49 to 320 ds | From 19 to 58 ds |
| | | 1 Pustular Eruption - - - - | 5 ms | Under cure date of last return |
| | 3 | 2 Exanthematous Eruption - | —— 90 to 240 ds | From 28 to 120 ds |
| | | 1 Exanthematous Eruption - | 298 ds | Under cure date of last return |
| | 2 | Tubercular Eruption - - - - - | —— 23 to 122 ds | From 26 to 35 ds |
| | 1 | Tubercular and scaly Eruption* | 19 ms | Under cure date of last return |
| | 3 | 1 Exanthematous Eruption with Sore Throat - - - - | Simultaneous with the Ulceration | 29 ds |
| | | 1 Exanthematous Eruption with Sore Throat - - - - | 4 weeks | 42 ds |
| | | 1 Exanthematous Eruption with Sore Throat - - - - | 59 ds | Under cure date of last return |
| | 2 | Tubercular Eruption with Sore Throat - - - - - - - - - - - | —— 75 to 91 ds | From 14 to 31 ds |
| | 1 | Papular, Scaly, and Tubercular Eruption, with Sore Throat* | 20 ms | Under cure at the date of last return |
| | 1 | Pustular and Tubercular Eruption with Sore Throat - - - | 68 ds | |
| | 3 | Sore Throat only - - - - - - - | —— 50 to 77 ds | From 43 to 63 ds |

* Primary Affections Cured by Mercury at a former Period.

To complete our paper on the subject, we shall here introduce the following important and highly interesting document circulated by the Army Medical Board among the surgeons of the army at large, and which cannot but be read with the deepest interest by every private practitioner.

(Copy) " *Army Medical Department,*
*2d April,* 1819.

" *Circular.*

" ON SYPHILIS.

" In transmitting the following summary of the conclusions on the question of syphilis and its treatment, we have to assure all that it may be considered as an unprejudiced statement drawn up from the answers *alone* of the regimental surgeons, to the queries transmitted by us to them in December last.

" *Without Mercury.*

" 1st. That since December 1816 to December 1818, there appears to have been treated for primary venereal ulcerations on the penis (including not only the more simple sores, but also a regular proportion of those with the most marked characters of syphilitic chancre, as described by Hunter and other writers) 1940 cases.

" 2d. That of these 1940 cases so treated, 96 have had secondary symptoms of different sorts.

" 3d. That in these 96 cases of secondary symptoms following sores treated without mercury, it was deemed necessary to have recourse to mercury for a cure for twelve of them; for which change, the following different reasons are assigned, in different cases, by the surgeons who treated them.

" *a.* On account of sloughing ulcers in the throat.
" *b.* The protraction of cure beyond the third week.
" *c.* Because the general health seemed to suffer.
" *d.* With a view of expediting the cure.
" *e.* The re-appearance of eruptions, or aggravation of symptoms.

" *Note.*—In several of these 12 cases, alterative doses of mercury were sufficient to effect the cure.

" 4th. That in 1940 cases of primary symptoms treated without mercury, (as described in par. 1st.) its use was resorted to in 65 of them; the reasons assigned being as follows:

" *a.* An indisposition to yield to the local application in three weeks.
" *b.* The sore spreading.
" *c.* The appearance of fresh sores.
" *d.* Buboes suppurating, and not disposed to heal.
" *e.* The general health appearing to suffer.
" *f.* A belief that the constitution became affected from the continuance of the sores.

" 5th. That these 1940 cases, treated, as here above stated, are now 'recovered of their venereal complaints,' and either doing their duty as soldiers, or have been discharged, for military reasons, totally unconnected with venereal disease.

" 6th. That the principal remedies employed have been (speaking in general terms, and with reference to primary sores) confinement to bed in many cases, in all to hospital, spoon diet, occasionally general bleeding when inflammation ran high, (in six or eight cases,) purgatives, antimonials, pretty generally emollient soothing applications in the first instance, generally cold or warm water, (the latter frequently injected between the prepuce and glans,) and the first externally applied, the water frequently mixed with the liquor plumbi; in the latter stages, the lotio hyd. submuriat. or muriat. in aqua calcis, lotio sulphat. cupri, argent. nitrat., &c. were employed. With reference to secondary symptoms, when mercury was not had recourse to, purgatives, antimonials, nitric acid, sarsaparilla, guaiacum in substance, or in combination with sarsaparilla, warm bath, nitro-muriatic acid bath, gargles when the throat is affected. In nodes, fomentations, scarifications, leeches, and blisters.

7th. That the average period required for the cure of primary symptoms without mercury, when bubo did not exist, has been twenty-one days; with bubo, forty-five days.

" 8th. That the average period for the cure of secondary symptoms without mercury has been from twenty-eight to forty-five days.

" 9th. That every man treated without mercury has been fit for immediate military duty on dismissal from hospital.

" *With Mercury.*

" 1st. That during the period specified before, there appears to have been treated of venereal ulcerations of the penis (the characters given of which do not appear to have been, in any essential degree, different from those treated without mercury) 2827.

" *Note.*—' It may be perhaps well to view these as more generally bearing the character of Hunter's chancre.'

" 2d. That of the 2827 thus treated and healed, 51 have had secondary symptoms.

" 3d. That there are good grounds for believing, that, in the majority of instances, when secondary symptoms have occurred, where the primary symptoms have been treated with mercury, that the secondary symptoms are more severe, and more intractable than when mercury had not been used for the primary sores.

" 4th. That one man treated by mercury for primary sores has been discharged the service on account of the injury his constitution sustained therefrom.

" 5th. That another man, after treatment for secondary symptoms by mercury, has been discharged the service in consequence of that complaint.

" 6th. That the average period occupied for the cure of primary symptoms without bubo, with mercury, has been thirty-three days; with bubo, fifty days; and that the great majority were fit for immediate military duty on dismissal from hospital.

" 7th. That the average period occupied in the cure of secondary symptoms has been forty-five days.

"*Note.*—'The treatment by mercury is so generally known, that it is deemed useless to describe it in either case." Much the same local applications were used in the treatment with mercury to the sores, as was described in that without it; perhaps more stimulating and escharotic applications were used, and less attention paid to regimen and diet, when mercury was given, at least less stress seems to have been laid on these.

"*General Observations.*

"1st. From the statement above made, it would appear that *all kinds of sores*, or primary symptoms of syphilis, may be cured (as far as a period of nearly two years will warrant the conclusion) without mercury.

"It is considered that the exceptions, noted in paragraph 4th, do not present valid objections to the above conclusions, on viewing the general testimonies on this point; but to the reasons there assigned for the necessity of having recourse to mercury, the most particular attention is required, as on these must the propriety or impropriety of that measure depend.

"2d. To guard against any fallacy in the comparative estimate of time employed in the cure of primary symptoms with and without bubo, it must be noticed that this is only an average statement; in some individual regiments the period required without mercury has been longer than that with mercury.

"3d. That it appears that the frequency or rarity of secondary symptoms would seem to depend on circumstances not yet sufficiently understood or explained, although the following fact would tend to the belief that either the constitutions of the men, or the mode of conducting the treatment without mercury, are the causes that possess the greatest influence in their production.

"In one regiment 4 secondary cases out of 24 treated without mercury supervened. In another regiment 68 cases have been treated within the specified time without mercury, all bearing marks of true venereal disease, (and 28 of these especially selected for their decided characters of chancre;) no secondary symptoms of any kind have hitherto made their appearance, and in all fifteen months have elapsed since they were treated.

"To this circumstance most particular attention is required, both with the view of ascertaining if peculiarity of constitution influences the appearance of secondary symptoms, and of pointing out the necessity of attending to the proper selection of local remedies adapted to the different stages and states of the sore, and to the general treatment of the constitution during the time the patients are in hospital, and that whether mercury be used or not.

"4th. That it appears that no peculiar secondary symptoms are seen to follow from peculiar primary sores.

"5th. It has been remarked, that, in cases healed without mercury, iritis has been frequently observed as a secondary symptom, in some instances by itself, in others attended with eruptions of

Page(s) missing

Page(s) missing different kinds. In these instances, mercury has been generally resorted to with success.

"6th. These appearances of primary ulcer, and repeated attacks of eruption, are the diseases which have been most frequently observed to succeed the non-mercurial practice.

"7th. The conclusions arrived at by the additional testimony of many more regiments not included in the number from whence this report has been drawn up, confirm, in every material circumstance, the results stated under both methods of treatment.

"From all that has been reported to us, we see no reason to stop the prosecution of the present inquiry, nor have we any objection to its being continued, but strictly in that spirit of patience, liberality, candour, and fidelity, that ought to characterize the inquiries after truth; a spirit altogether remote from the precipitancy of innovation, the acrimony of disputants, or the stickler for any particular doctrine.

"1st. It is therefore desired, that the queries heretofore submitted, with these additional points left undecided in this letter, may be considered as the leading objects for consideration in the future prosecution of the subject.

"2d. That every syphilitic case, whether secondary or primary, be duly entered in the register, with full description of the characters of the sores, symptoms, and treatment, so that the results of each half year may be distinctly and clearly stated in the reports required on these occasions; that every man belonging to any regiment, treated in a different regimental hospital to his own, shall invariably be reported through this office to his own regimental surgeon, who will duly register the report, and at the half yearly periods, state the results.

"That it is essentially necessary that each regimental surgeon keep a watchful eye over all men treated without mercury, and frequently examine them, and that whenever answers are required to these queries at a future period, which they will, (say the 1st. January 1820,) the state of the men now reported shall at that time be distinctly referred to, in the same tabular form as was required by the late queries, commencing as before from 20th December 1816.

"We wish it to be distinctly understood, that we do not enforce the non-mercurial plan of treatment in any case, still farther is it from our wish to incur any unnecessary risk or danger to the soldiers, by unnecessary detention from duty, from a protracted treatment without mercury, in those cases where it has been begun. At all times, this is left to the discretion of the surgeon, who, we are persuaded, will act in the most conscientious manner for the good of his patient, and the interests of the service.

(Signed) 'J. M'Grigor,

'W. Francklin.'"

Dr. Hennen here enters into a vindication of the non-mercural practice as far as regards the recent objection made by Dr. Hamilton, jun. who "conceives that very alarming consequences are likely to arise to children yet unborn, from the practice of the army surgeons." It is well known, as Dr. Hennen remarks, that there are various opinions on the subject of the venereal disease of the fœtus in utero; some contending that the *mother*, not the father, communicates the disease to the embryo; others, that it is the father, not the mother; a third party contend that the infant may be affected by both father and mother; while John Hunter denies the possibility of the fœtus in utero being affected by either. Impressed with the difficulty of forming a decided judgement upon so obscure a subject, Dr. Hennen first states his speculative opinions, and then details such facts as he has been able to collect.

"Unless a man has primary symptoms himself, I apprehend it is physically impossible for him to communicate primary symptoms to a female. Unless a female has primary symptoms, I hold it equally mpossible for her to have secondary symptoms; and except she has secondary symptoms, she, I apprehend, cannot communicate them to the children in her womb; she may, indeed, as we all know, communicate primary sores to the fœtus in its passage through her vagina." 550.

Numerous instances, Dr. H. observes, are on record, where women having secondary symptoms, bring forth healthy children; and where fathers who have long had secondary affections beget perfectly sound offspring.

"That children are born with a disease *supposed* to be syphilis, and that this disease is not only fatal to them, but can be communicated by them to their nurses, and be propagated by the nurse to the destruction of more lives, is a fact that no man can pretend to deny. The nurse, however, must have a primary sore on her nipple or elsewhere, before she can disease the child. I know it to be a positive fact, that a nurse with secondary symptoms may suckle children with perfect impunity to them."

We ourselves have lately seen some children born with all the appearances of syphilitic defœdations, which defœdations disappeared by mild aperients without any mercury. This, indeed, does not prove that they were but syphilitic affections *in appearance;* it only goes to prove, that these congenital diseases are curable without mercury. The above facts render it extremely doubtful from which of the parties the disease is transmitted; or, indeed, whether it is transmitted by either. Dr. Hennen having circulated a string of queries in Scotland and the northern district of England, relative to this subject, the result is given in a table. The total of children born of parents who had undergone the non-mercurial treatment was thirteen, eleven of whom were born healthy, two were still-born, and one has become unhealthy since birth. A woman in the 88th Regt. excited considerable attention in the Ed. Castle Hospital, in consequence of being diseased and pregnant. At the time that the secondary symptoms appeared in her, she was three months gone. She was treated without mercury, and the disease gave way in a little more than three months and a half. She was delivered of a still-born child in the eighth month of utero-gestation, not having felt any movement of the fœtus for two months before. Another woman treated without mercury also aborted of a dead child at the end of the seventh month. In both cases there was considerable abrasion of the cuticle upon the arms and sides, but no distinct eruption. These two cases certainly go towards the corroboration of Dr. Hamilton's doctrine; but two cases can prove little.

In the conclusion of this part of Dr. Hennen's work, he states, from good authority, that the non-mercurial practice has been adopted by some of the naval surgeons with a success even greater than in the army. In one ship fifty patients were treated without mercury, in one only of whom did secondary symptoms appear, under the form of blotches, and they were entirely removed in the course of eight days, without any mercury internally or externally applied. Other testimonies also, from the officers of the army, have recently come to the knowledge of our author, "which still farther tend to confirm the principles which he has advocated."

We have now laid a full analysis of this important section of Dr. Hennen's publication before our brethren, which will enable them to judge for themselves respecting the principles and practices therein brought forward.

We have but few remarks of our own to make on this momentous question. We conceive that the *possibility* of curing both primary and secondary syphilis is now beyond the shadow of doubt; and for the ascertainment of this important point we are solely indebted to the Army Medical Board and Officers, who alone could have made the trial on a scale, and with a precision that must carry conviction to the most sceptical mind. For this service to Medical Science posterity will long record the names of her zealous cultivators, in the British army; nor will their liberal cotemporaries withhold the meed of their well-earned approbation.

In respect to the *elegibility* of treating syphilis without mercury, we think that reasonable doubts may be entertained. While the knowledge disclosed in the foregoing documents imperiously indicates the propriety of a far greater restriction in the use of mercury, for the cure of syphilis, than we have hitherto thought proper, so there is nothing in the said documents that fairly goes to contra-indicate a moderate administration of the old remedy, where scrofula, idiosyncrasy of constitution, or other reason do not stand in the way, and induce us to trust to the powers of Nature, assisted by rest, abstinence, and gentle evacuations.

To what we have said of Dr. Hennen's work in general, at the opening of this article, we can add nothing more than our full conviction that it is one of the most practical, useful, and interesting publications which have fallen under cognizance for many years past.

